# Successful Management of Steroid‐Refractory Immune‐Related Pneumonitis With Mycophenolate Mofetil: A Case Report

**DOI:** 10.1002/rcr2.70180

**Published:** 2025-04-21

**Authors:** Hiroaki Ota, Daichi Fujimoto, Yoshiki Negi, Takashi Murata, Mayuko Tokuda, Tomoki Higashiyama, Akio Tada, Toshiyuki Minami, Taiichiro Otsuki, Koji Mikami, Ryo Takahashi, Kozo Kuribayashi, Takashi Kijima

**Affiliations:** ^1^ Department of Respiratory Medicine and Hematology Hyogo Medical University Nishinomiya Hyogo Japan

**Keywords:** immune‐related pneumonitis, mycophenolate mofetil, steroid‐refractory pneumonitis

## Abstract

Steroid‐refractory immune‐related pneumonitis is a clinical challenge with limited evidence‐based treatment strategies. Current guidelines recommend the use of immunosuppressants; however, the optimal type and dosage of these agents remain unclear. Herein, we report a case of steroid‐refractory immune‐related pneumonitis that was successfully treated with mycophenolate mofetil (MMF). The patient did not respond to high‐dose steroid therapy as initial treatment but showed significant improvement in both subjective symptoms and imaging findings after the additional administration of MMF. Subsequent tapering of the MMF dose led to worsening imaging findings, which improved upon re‐escalation of the MMF dose. This case highlights the potential efficacy of MMF for the treatment of steroid‐refractory immune‐related pneumonitis and provides valuable insights into the administration of MMF and its potential role in managing similar cases.

## Graphical abstract

Herein, we report the case of an 82‐year‐old male with pleural mesothelioma who developed steroid‐refractory immune‐related pneumonitis after treatment with ipilimumab and nivolumab, which was successfully managed by mycophenolate mofetil treatment.

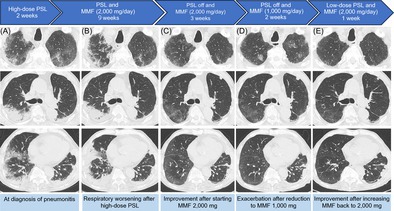

## 1 Introduction

Immune checkpoint inhibitors (ICIs) have revolutionised cancer therapy [1]; however, they can cause immune‐related adverse events (irAEs), including pneumonitis, which affects approximately 5%–19% of patients [2, 3].

Corticosteroids are the standard treatment for immune‐related pneumonitis [4, 5]; however, some cases, termed steroid‐refractory, do not respond. Although guidelines recommend the use of additional immunosuppressants for steroid‐refractory cases, the best treatment approach has yet to be firmly established, highlighting the need for further research to guide clinical decision‐making [4, 5].

Herein, we report the case of an 82‐year‐old male with pleural mesothelioma who developed steroid‐refractory immune‐related pneumonitis after treatment with ipilimumab and nivolumab, which was successfully managed by MMF treatment.

## 2 Case Report

An 82‐year‐old male diagnosed with epithelioid pleural mesothelioma (cT1N0M0) was referred to our hospital; a first‐line treatment with ipilimumab and nivolumab was initiated. After eight courses of these therapies, follow‐up computed tomography (CT) revealed infiltrative shadows in both lungs. Clinically, the subjective symptoms observed in this case were fever and dyspnea. Blood tests revealed elevated CRP and SP‐D levels, while KL‐6 remained within the normal range. Sputum cultures, procalcitonin, and SARS‐CoV‐2 polymerase chain reaction were negative. Given the imaging findings, along with the patient's medical history and physical examination findings, a differential diagnosis was considered. Connective tissue disease‐associated interstitial lung disease (ILD) and vasculitis‐associated ILD were deemed unlikely based on the negative autoantibodies results in serological testing, including anti‐nuclear antigen antibodies, anti‐aminoacyl‐tRNA synthetase antibodies, anti‐cyclic citrullinated peptide antibodies, anti‐Sjögren's‐syndrome‐related antigen A antibodies, anti‐Sjögren's‐syndrome‐related antigen B antibodies, and anti‐neutrophil cytoplasmic antigen antibodies. Additionally, hypersensitivity pneumonitis was considered unlikely due to the absence of any identifiable history of antigen exposure. The patient discontinued immunotherapy at the time of pneumonitis identification and was subsequently treated with lascufloxacin, followed by tazobactam/ceftolozane, but showed no improvement in oxygen saturation and worsening respiratory status, requiring an increase in oxygen support to 2 L/min. Based on these clinical findings, ICI‐induced pneumonitis (Grade 3) was suspected. Methylprednisolone (1000 mg/day) was administered for 3 days, followed by prednisolone at 60 mg/day (1 mg/kg/day) for 8 days. However, radiographic findings taken after these treatments showed a persistent infiltrative shadow, and the patient required increased oxygen support, with the flow rate rising from 2 to 4 L/min. Subsequently, a second course of methylprednisolone (1000 mg/day) was administered for 3 days. However, there were no significant changes in the respiratory status of the patient.

To conduct further investigation, bronchoscopy was performed 1 week after the start of the second course of methylprednisolone (1000 mg/day) followed by prednisolone at 40 mg/day. Endobronchial examination revealed no sputum retention. Bronchoalveolar lavage (BAL) fluid analysis showed a neutrophil proportion of 29%, a lymphocyte proportion of 58%, and a macrophage proportion of 13%. The proportions of monocytes, eosinophils, and basophils were all 0%. The CD4/CD8 ratio was 0.33. No specific cell profiles were observed. Pathological examination of the trans‐bronchial lung biopsy specimen revealed no malignant findings, and culture tests of the lower respiratory tract specimens obtained during bronchoscopy were negative for pathogens. Based on these findings, the patient was diagnosed with steroid‐refractory ICI‐induced pneumonitis. This led to the decision to initiate immunosuppressive therapy using MMF (2000 mg/day). Thereafter, the patient's respiratory condition improved, and a CT performed 2 weeks after MMF initiation showed remission of the infiltrative shadow. The steroids were tapered and stopped 14 weeks after initiation, and the patient was transitioned to MMF monotherapy.

**FIGURE 1:**
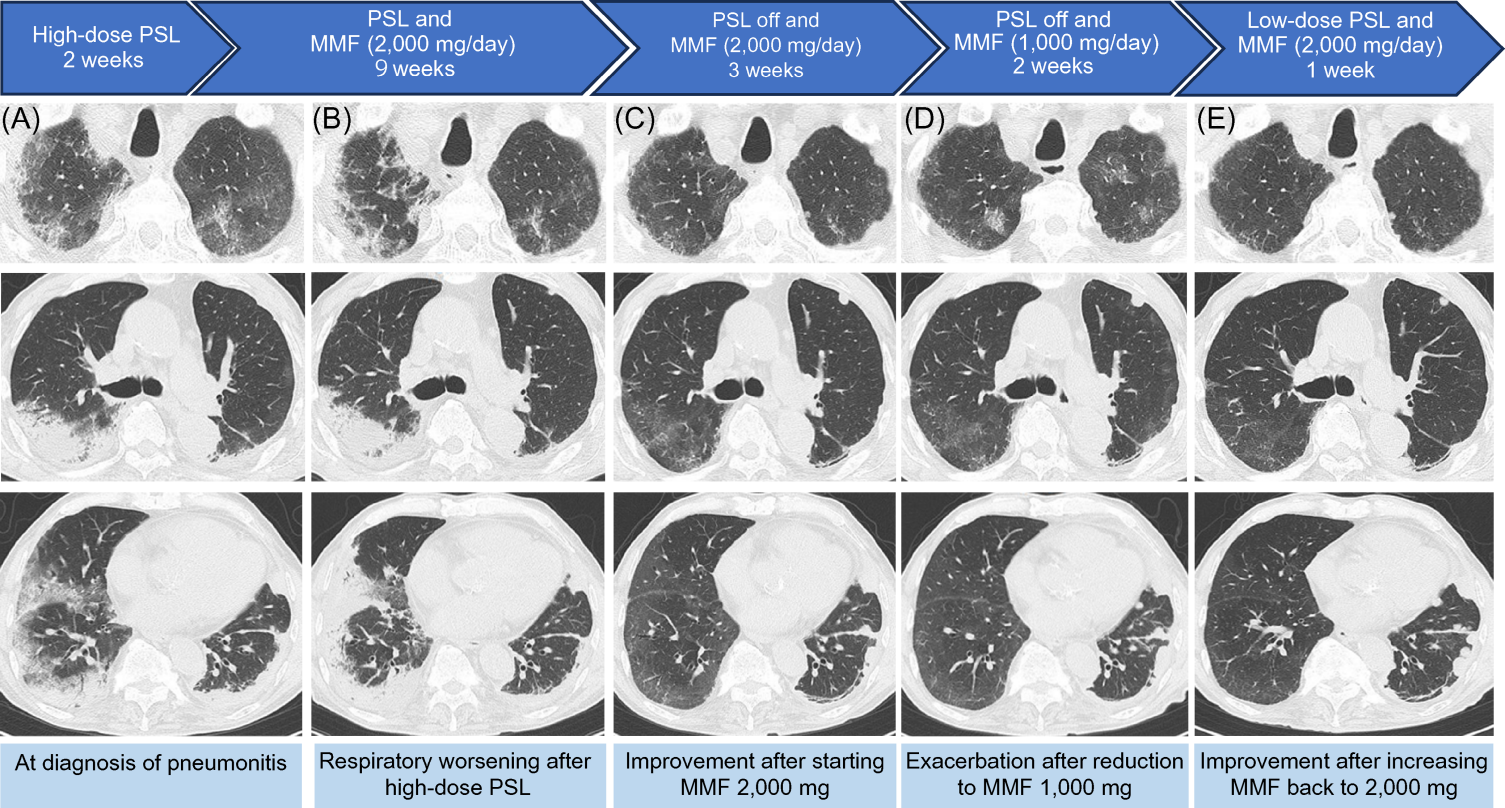
Treatment details and duration for pneumonitis with changes in chest computed tomography are presented (A) at the time of pneumonitis diagnosis; (B) at time of respiratory worsening after high‐dose PSL; (C) start of MMF 2000 mg showing some improvement; (D) after reduction to MMF 1000 mg showing exacerbation of symptoms; and (E) after increasing the dose back to 2000 mg showing improvement. MMF, mycophenolate mofetil; PSL, prednisolone.

After careful monitoring for 4 weeks following the transition to MMF monotherapy to ensure no recurrence of pneumonitis after steroid discontinuation and no signs of deterioration, the MMF dose was reduced to 1000 mg/day. However, chest radiography and CT performed 2 weeks after the reduction of MMF showed new ground‐glass opacities in the bilateral lungs. The patient was administered levofloxacin without improvement. It was suspected that the reduction of the MMF dose triggered a flare‐up of the immune reaction and induced the recurrence of pneumonitis. The dose of MMF was increased to 2000 mg/day in combination with prednisolone at 30 mg/day. CT scans showed rapid improvement in the shadows 7 days after switching to this treatment. The patient has been under follow‐up for 3 months on MMF 2000 mg/day, during which prednisolone was tapered, showing stable respiratory function without any further worsening of pneumonitis. The imaging findings during the course of pneumonitis are shown in Figure 1.

## 3 Discussion

The treatment of patients with steroid‐refractory immune‐related pneumonitis is challenging. Given the limited evidence, current guidelines recommend supplementing steroid therapy with immunosuppressants for this condition. Furthermore, a recent report has indicated a high frequency of worsening symptoms, particularly in cases of grade 3 or higher, when steroids are used as monotherapy, highlighting the critical need for the addition of immunosuppressants [6]. In our case, the administration of MMF led to clinical improvement and was considered to have contributed to sustained improvement thereafter. Considering these insights and the favourable response to MMF, immunosuppressants should be considered early, particularly in refractory cases.

The diagnosis of drug‐induced pneumonitis cannot be definitively confirmed but is based on clinical assessment, including patient history, physical examination, laboratory tests, and imaging findings. The diagnostic criteria include newly identified pulmonary parenchymal opacities on imaging, a temporal association between symptom onset and the initiation of a systemic therapeutic agent, and the exclusion of other likely causes [7]. Although a comprehensive exclusion of viral infection was not feasible in this case, the clinical course, including the rapid improvement of pneumonitis following MMF administration and its recurrence upon dose reduction, suggested that a viral aetiology was unlikely. Generally, bronchoscopy, including BAL, carries risks. In this case, the patient's condition continued to deteriorate despite steroid therapy. Given this worsening, the potential benefits of BAL were considered to outweigh the risks. Therefore, BAL was subsequently performed, which provided critical findings that allowed us to confirm the diagnosis of steroid‐refractory immune‐related pneumonitis.

The European Society for Medical Oncology guidelines recommend several immunosuppressants, including MMF, based on expert opinion, whereas the American Society of Clinical Oncology guidelines do not mention MMF [4, 5]. MMF selectively targets lymphocytes, making it a promising treatment for irAEs in which lymphocytes play a central role [8]. In addition to its anti‐inflammatory effects, MMF has antifibrotic properties, positioning it as a potential alternative to steroids for treating pneumonitis that may progress to fibrosis during inflammation [9]. Based on these considerations, MMF was chosen due to its therapeutic efficacy and the advantage of it being an oral agent, which facilitates continued outpatient management. Given these findings and the favourable response observed in our case, MMF demonstrates value as a reasonable and potentially advantageous choice among immunosuppressants for the management of steroid‐refractory pneumonitis.

Patients with steroid‐refractory immune‐related pneumonitis are challenging to treat. Though there have been several cases of patients who received MMF in this condition [10, 11, 12], the optimal dose and schedule remain unclear, leaving practical guidance unspecified. Notably, in the present case, pneumonitis was effectively controlled using MMF at 2000 mg/day but relapsed when MMF was reduced to 1000 mg/day, suggesting that adequate dosing may be critical for disease control and that insufficient dosing could lead to recurrence. The optimal MMF dosage is still under debate, and previous studies have reported that the recommended dosage for systemic sclerosis‐associated ILD ranges from 2000 to 3000 mg [13]. Based on these findings, a dosage of 2000 mg was considered appropriate in this case. Furthermore, as observed in drug‐induced pneumonitis cases, similar to corticosteroids, MMF may exhibit dose‐dependent efficacy, where lower doses are effective in some instances, whereas in others, a higher dosage is required for sufficient therapeutic response. Given the limited availability of clinical trial data, successful case reports are essential for developing effective management strategies. This case report provides valuable insights into the administration of MMF and its potential role in the management of similar cases.

In conclusion, we encountered a case of steroid‐refractory ICI‐induced pneumonitis that showed a significant response to MMF. This case adds to the growing body of evidence supporting the use of MMF in the management of steroid‐refractory pneumonitis and underscores the need for further research in this area.

## Author Contributions

All the authors contributed to the conception of the study. A literature search was performed and the first draft of the manuscript was written by Hiroaki Ota. The manuscript was edited by Daichi Fujimoto. All authors commented on the previous versions of the manuscript. All the authors have read and approved the final version of the manuscript.

## Ethics Statement

The authors declare that appropriate written informed consent was obtained for the publication of this manuscript and the accompanying images.

## Conflicts of Interest

The authors declare the following financial interests/personal relationships, which may be considered potential competing interests: Daichi Fujimoto has received grants and personal fees from AstraZeneca KK and Boehringer Ingelheim Japan Inc., as well as personal fees from Ono Pharmaceutical Co. Ltd., Bristol‐Myers Squibb Co. Ltd., Taiho Pharmaceutical Co. Ltd., Chugai Pharmaceutical Co. Ltd., MSD KK, Eli Lilly Japan KK, Novartis Pharma KK, Kyowa Kirin Co. Ltd., and Janssen Pharmaceutical KK, outside of the submitted work. He also participates on an advisory board for AstraZeneca KK and Chugai Pharmaceutical Co. Ltd. Taiichiro Otsuki has received grants and personal fees from AstraZeneca, Bristol Myers Squibb Co, Chugai‐Roshe, TAIHO Pharmaceutical Co, Daiichi Sankyo Company Ltd, Takeda Pharmaceutical Company, MSD KK, and ONO Pharmaceutical Co, outside of the submitted work. Toshiyuki Minami has received grants and personal fees from Chugai Pharmaceutical, Bristol Myers Squibb, AstraZeneca, KYORIN Pharmaceutical, MSD, and TAIHO Pharmaceutical, outside of the submitted work.

## Data Availability

The data that support the findings of this study are available from the corresponding author upon reasonable request.
